# Pre-thrombectomy cerebral edema affects outcomes in acute stroke patients treated with thrombectomy

**DOI:** 10.7150/ijms.105692

**Published:** 2025-04-28

**Authors:** Lu Yang, Yuan Kan, Changhong Ren, Sijie Li, Chuanhui Li, Longfei Wu, Jiali Xu, Wenting Guo, Haiqing Song, Qingfeng Ma, Wenbo Zhao, Xunming Ji

**Affiliations:** 1 Department of Neurology, Xuanwu Hospital, Capital Medical University, Beijing, China.; 2 Department of Neurosurgery, Xuanwu Hospital, Capital Medical University, Beijing, China.; 3 Beijing Key Laboratory of Hypoxic Conditioning Translational Medicine, Xuanwu Hospital, Capital Medical University, Beijing, China.; 4 Center of Stroke, Beijing Institute of Brain Disorder, Capital Medical University, Beijing, China.; 5 Department of Rehabilitation Medicine, Beijing Shijitan Hospital, Capital Medical University, Beijing, China.; 6 Center for Rehabilitation Medicine, Department of Neurology, Zhejiang Provincial People's Hospital, Hangzhou Medical College, Hangzhou, Zhejiang, China.

**Keywords:** ischemic stroke, thrombectomy, net water uptake, cerebral edema

## Abstract

**Objective**: Cerebral edema significantly impacts the functional outcomes in patients with acute stroke treated with thrombectomy, especially those with an extended time window (6-24 hours). This study was to investigate whether pre-thrombectomy cerebral edema predicts functional prognosis in ischemic stroke patients within an extended onset time window.

**Methods:** All patients from Xuanwu Hospital of Capital Medical University underwent computed tomography (CT) examination and endovascular treatment between December 2021 and December 2023. Quantitative Net Water Uptake (NWU) was assessed according to baseline CT. The ability to predict onset time and outcomes was assessed by univariate receiver operating characteristic curves and logistic regression analyses. The primary endpoint was an unfunctional outcome at 90 days, defined as a modified Rankin Scale Score of 3-6.

**Results:** We reviewed a total of 247 patients, and the last 134 were included in the study, of whom 41.8% had stroke onset within 6 hours. NWU was significantly lower in patients with stroke onset within 6 hours (6.57±3.43) compared to 6-24 hours (11.69±3.01). Of patients with onset times of 6-24h, the area under the curve (AUC) for distinguishing patient groups according to NWU% was 0.863, with a cut-off value of 9.3 (sensitivity, 80.8%; specificity, 82.1%). A multivariable predictive model including NWU% and age yielded the highest diagnostic ability, with an AUC of 0.857 (sensitivity, 66.7%; specificity, 92.9%).

**Conclusion:** NWU as an imaging biomarker of brain edema predicts functional prognosis after endovascular recanalization therapy in ischemic stroke patients within an extended onset time window.

## 1. Introduction

Endovascular treatment is currently the primary treatment strategy for acute ischemic stroke (AIS)^1^, but endovascular treatment of AIS still has several limitations. First and foremost, treatment efficacy needs to be improved; currently, even among patients in whom the blood vessel is successfully recanalized, only 51% achieve good outcomes^2^, ^3^. In clinical practice, the treatment process is complex, particularly for patients with a stroke onset time within 6-24 hours when advanced imaging is still required. In a systematic review of seven observational cohorts, Diestro et al. found no statistically significant differences between no-perfusion and perfusion computed tomography (CT) for long-term clinical and symptomatic intracerebral hemorrhage (sICH) in the late window of emergent large-vessel ischemic stroke^4^.

Cerebral edema is a common cause of severe neurological impairment and death due to acute brain injury^5^. A recent study showed that approximately 30% of patients with ischemic stroke have varying degrees of cerebral edema following successful recanalization^6^. Therefore, how to accurately assess the degree of cerebral edema and predict the prognosis of thrombolysis is critical to endovascular recanalization treatment decisions and outcomes. More recently, it has been observed that the effects of successful recanalization are mediated by infarct growth and edema reduction^7^, ^8^.

However, there remains a gap in quantitatively identifying brain edema using neuroimaging. It is thus necessary to identify neuroimaging markers that can identify brain edema early, with high sensitivity and specificity. The Alberta Stroke Program Early Computed Tomography Score (ASPECTS) was derived by assessing the degree of hypoattenuation of the ischemic tissue relative to normal tissue^9^. Net Water Uptake (NWU) is an imaging index that uses the change in CT density (low attenuation) of the ischemic brain tissue to quantify the severity of ischemic brain edema after stroke^10^, ^11^. An increasing number of studies have shown that NWU can be used to estimate the onset time of stroke, which has been described as an important biological marker of brain edema, as well as an individual indicator of the "tissue clock" in AIS patients^12^. More importantly, NWU can be calculated based on NECT, a parameter that can be quickly obtained in primary hospitals^13^. NWU directly reflects the low degree of attenuation but has not been used as an imaging marker for the selection of thrombolysis or thrombectomy treatment for stroke patients. Therefore, we need more studies to validate the value of NWU for clinical use.

The purpose of this study was to investigate whether NWU as an imaging biomarker of brain edema predicts functional prognosis after endovascular recanalization therapy in AIS patients within an extended onset time window.

## 2. Material and methods

### 2.1 Patients

Patients with acute cerebral infarction in the anterior circulation who underwent vascular recanalization at Xuanwu Hospital of Capital Medical University between December 2021 and December 2023 were retrospectively enrolled. All patients had a known time of onset of witnessed stroke. Patients were divided into two groups according to the time from stroke onset to admission: within 6 hours and 6-24 hours. Stroke onset time was defined as the time from symptom onset to admission. The inclusion criteria were as follows: (1) age > 18 years; (2) acute middle cerebral artery cerebral infarction with mechanical thrombectomy; (3) multimodal CT including NECT, Computed Tomography Angiography (CTA), and Computed Tomography Perfusion (CTP); and (4) known NIHSS score at admission and mRS score at 90 days of follow-up. The primary endpoint was an unfavorable functional outcome, defined as an mRS Score of 3-6. The exclusion criteria were as follows: (1) posterior circulation infarction; (2) previous intracranial hemorrhage, brain surgery, or large-area lesions; (3) failure of thrombectomy, final digital subtraction angiography (DSA) showing no successful recanalization, or angiography 24 h after the operation showing no recanalization; (4) ASPECTS software showing segmentation errors due to bus deviation or posture error without basic influence data; and (5) patients lost to follow-up. Successful recanalization was defined as modified thrombolysis with cerebral infarction (mTICI) score of 2b-3^14^. Some patients with large core infarcts included in our study met the clinical evaluation criteria for vascular resection; they were functionally independent before onset, had this onset within 24 hours, and demonstrated potentially disabling neurologic deficits. They also had a CTA-confirmed occlusion of a large anterior circulation artery that was the cause of the current cerebral infarction and a CTP-assessed infarct volume of 70-100 ml.

### 2.2 Imaging protocol

All patients underwent 256-slice spiral CT prior to mechanical thrombectomy (GE, OPTIMA CT680, USA), as well as a CTP. First, the conventional head CT scan was performed, with a slice thickness and spacing of 5 mm. Whole-brain CTP was performed with a tube voltage of 70 kV and tube current of 100 mA. All patients were injected with a high-pressure syringe through the elbow vein or forearm vein with 40 ml iopromide (370 mg/ml; Bayer Pharmaceuticals, Germany) immediately after 40 ml isotonic saline was injected at a speed of 6 ml/s in both groups. Injection Scanning began in the last 4 s and lasted for 60 s. All post-processing of raw perfusion data and image analyses of raw CT and CTP were performed by radiologists at Xuanwu Hospital.

### 2.3 Imaging analysis

The NWU is an imaging index that uses the change in CT density (low attenuation) of ischemic brain tissue to quantify the severity of ischemic brain edema after stroke^15^. Ischemic brain tissue with marked hypoattenuation on NECT was confirmed visually. We used automated ASPECTS instead of CTP to assess the ischemic regions and calculate the NWU, which is more readily available and accessible. An automated software-based analysis (syngo. via Research Frontier, ASPECT Score-Tool v2.0.3; Siemens Healthineers) was used to calculate mean Hounsfeld unit (HU) values for ten regions of affected ASPECTS regions on non-contrast CT images and expressed as a percentage of the HU difference^16^. Using a predefined threshold for the relative HU difference, each ASPECTS region in the ischemic hemisphere was classified as affected (ischemic changes detected using software) or unaffected. The region of the ROI used to measure the density of ischemic (dischemic) and normal (normal) tissue was set between 20 and 80 Hu^17^. NWU% was calculated according to the following equation: NWU% = (1-Dischemic/Dnormal) ×100. The NWU% of the ischemic tissue was evaluated and validated by two experienced neuroradiologists (Figure 2).

### 2.4 Statistical analysis

Kolmogorov-Smirnov tests were used to determine whether the datasets were well modeled by a normal distribution. Normally distributed data are presented as the mean ± standard deviation (SD) and non-normal distribution data are presented as the interquartile range (IQR). Categorical variables are presented as counts and percentages. Receiver operating characteristic (ROC) curves were used to determine the discriminatory power and optimal cut-off values of NWU%. To explore the potential predictors of 90-day mRS, univariate binary logistic regression analysis was applied to analyze the association between the 90-day mRS score and the selected variables. Candidate variables with a p-value of less than 0.1 were drawn into corresponding multivariable binary logistic regression models using the method of conditional forward, retaining in the final model including only those that were specified prior, or that had an independent relationship with 90-day mRS. All statistically significant differences were accepted at a p-value of < 0.05. All statistical analyses were performed using SPSS version 26 (IBM Corp., Armonk, NY, USA).

## 3. Results

### 3.1 Baseline characteristics of the study population and patient selection

**Figure 1:**
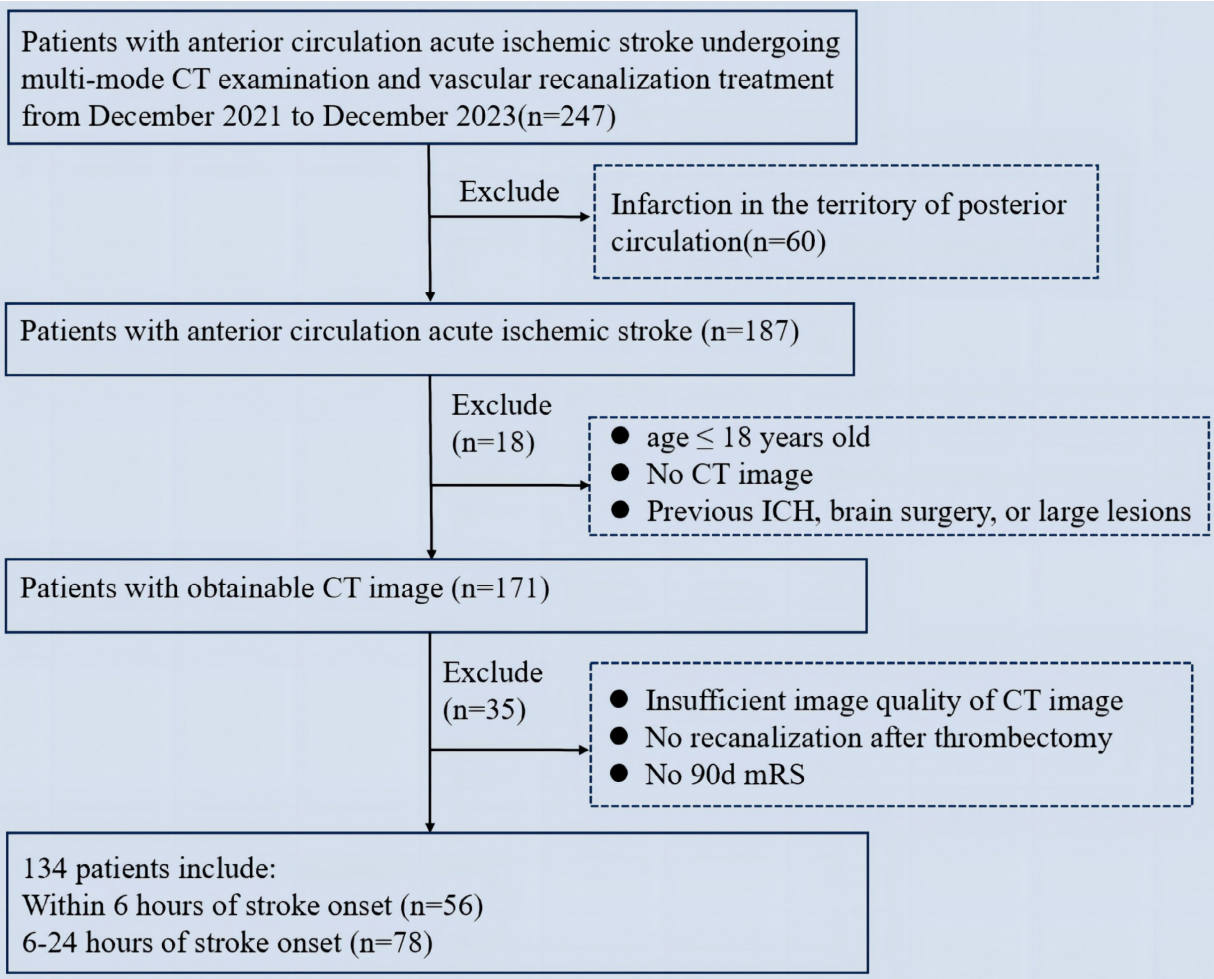
Flowchart of patient selection. ICH: Intracranial hemorrhage; mRS: modified Rankin Scale.

**Figure 2:**
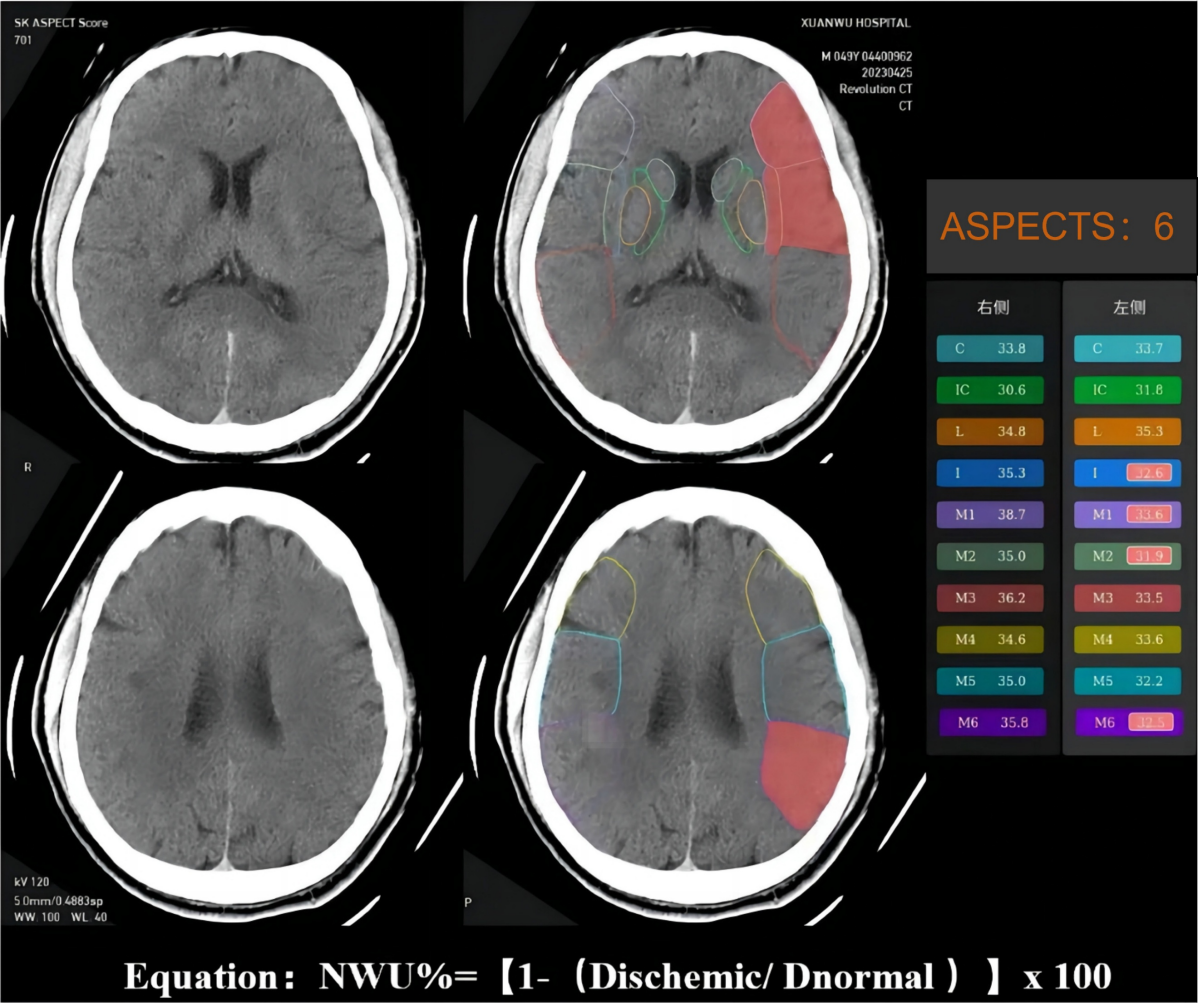
Example for illustrating how the CT-ASPECTS-NWU% value was calculated. Density ischemic was 32.65 (the mean Density of ischemic ASPECTS regions [I, M1, M2, M6]). The Density normal was 36.2 (the mean HU of normal ASPECTS regions [I, M1, M2, M6]). The CT-ASPECTS-NWU% value was 9.81%.

We retrospectively enrolled 247 patients with stroke who underwent mechanical thrombectomy. The clinical and imaging characteristics of the study population are presented in Table 1. A total of 134 patients met the inclusion criteria (Figure 1). Of the 134 patients who met the inclusion criteria, 56 (41.8%) presented within 0-6 hours, and 78 (58.2%) presented within 6-24 hours. The two groups of patients (0-6 and 6-24 hours) were comparable in terms of age (mean standard deviation, 59.2 ± 12.45 years vs. 63.0 ± 13.78 years, p=0.196), gender (female, 32.1% vs. 32.1%, p=0.982), and the median NIHSS score admission was 14 (IQR 10-17) minutes and 15 (IQR 10-19) minutes, respectively. Comorbidities included hypertension (66.1% vs. 61.5%, p=0.729), diabetes mellitus (19.6% vs. 29.5%, p=0.008), and hypercholesterolemia (16.1% vs. 23.1%, p=0.025). The median time from stroke onset to admission was 264.39 (IQR 276-408.5) minutes and 621.17 (IQR 507-820) minutes, respectively.

Sixteen patients (28.6%) were treated with bridging therapy (pretreatment with intravenous thrombolysis before mechanical thrombectomy), while 39 (69.6 %) and 63 (80.8 %) patients in the two groups were treated with tirofiban after mechanical thrombectomy, respectively. The median time from onset to reperfusion (OTR) in the two groups was 337.41 (IQR 276-408.5) minutes and 698.99 (IQR 507-820) minutes, respectively. Patients presented with median NIHSS scores of 14.88 (IQR 10-19) vs. 13.27 (IQR 9-18), mean CTP mismatch 102.60 ml (± 54.92 ml) vs. 96.36 ml (± 70.68 ml), a median CBF<30% 25.73 ml (IQR 3.60-44.65 ml) vs. 16.02 ml (IQR 0.5-20.7 ml) in the 0-6 hours and the 6-24 hour groups, respectively. Vascular occlusions were primarily located in the M1 segment of the middle cerebral artery. There were five sICH in the 0-6 hours group and six sICH in the 6-24 hours group. The percentages of patients with favorable functional outcomes at 90 days were 67.9% and 51.3%, respectively.

### 3.2 Prediction models of unfavorable functional outcome (mRS 3-6) in 6-24h group

Next, we focused on risk factors for 90-day unfavorable functional outcomes with stroke onset time within 6-24 hours. Relevant parameters were included in multiple multivariate models, considering collinearity. In the univariate analysis, age (p=0.014), NWU% (p<0.001), ASPECTS (p<0.001), and onset time (p<0.001) were found to be significantly associated with the 90-day mRS (3-6). In contrast, the multivariate regression analysis showed that age (p=0.041), NWU% (p=0.036), and ASPECTS scores (p=0.011) were independent influencing factors of 90-day mRS (Table 2).

### 3.3 ROC-curve for predicting 6h onset time point and 90-day unfavorable functional outcome (mRS 3-6)

**Figure 3:**
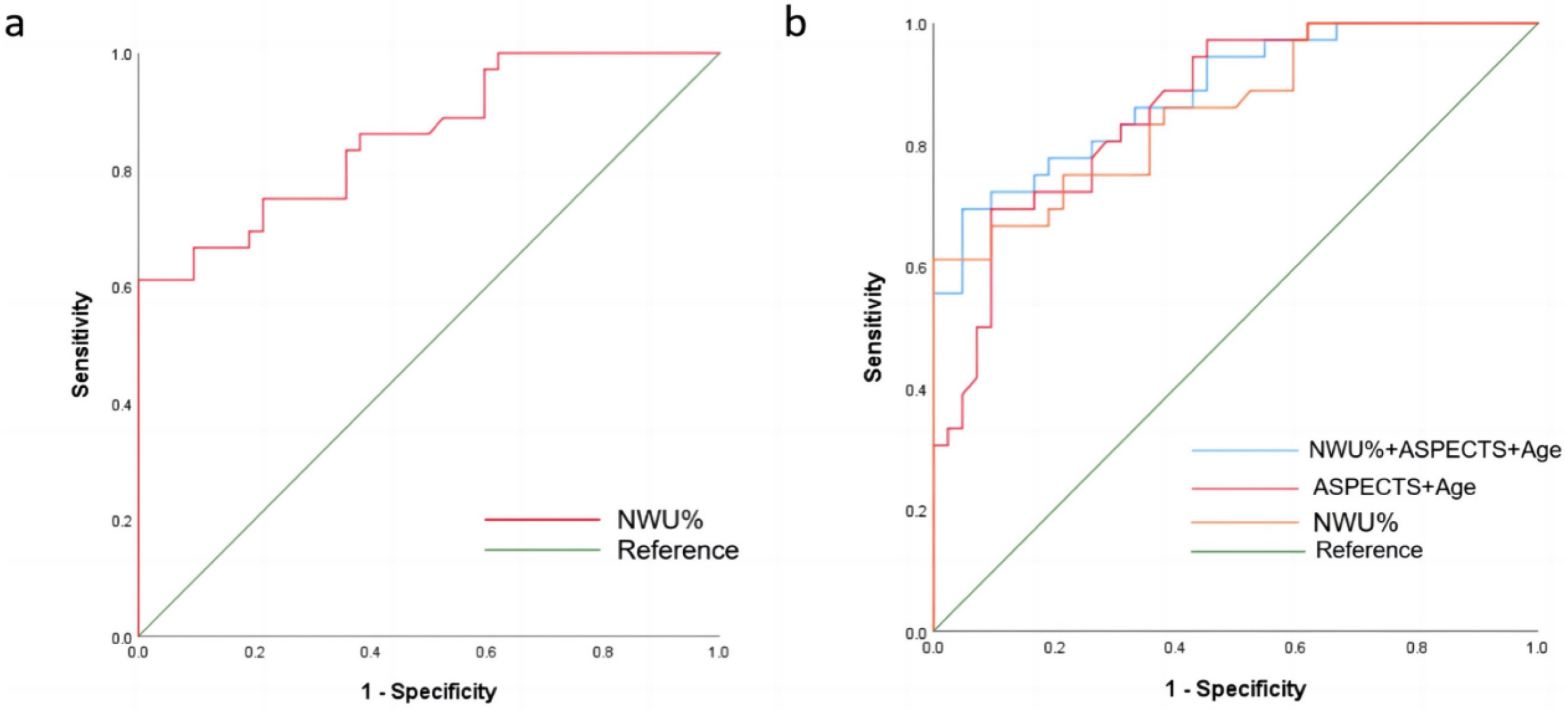
ROC curve analysis for identifying the ideal cut-off value of the NWU% for pr).edicting 6h onset time point and 90-day unfavorable functional outcome (mRS 3-6).

The mean of NWU% for patients within 6 hours was 6.57 (95% CI, 5.65 to 7.49), while that for patients with stroke onset time within 6-24 hours was 11.69 (95% CI, 11.01% to 12.37%). When classifying stroke onset to admission time within 6 hours and 6-24 hours, the ROC curve comparison showed the highest diagnostic performance for NWU%, with an AUC of 0.863 (95%CI 0.800-0.927, p< 0.05). The ideal cutoff value was 9.3, which had a sensitivity of 80.8% and a specificity of 82.1% (Figure 3a, Table 3). Multivariate logistic regression analysis of all significant factors (NWU%, and age) was performed to predict the probability of a favorable outcome at 90 days, compared with the NWU% ROC curve analysis. The AUC of NWU% was 0.856 (95% CI 0.774-0.939, p < 0.05, sensitivity 61.1%, specificity 100%), and the optimal cut-off value for classifying the 90-day unfavorable outcome was 13.3. A multivariable predictive model including NWU% and age yielded the highest diagnostic ability, with an AUC of 0.857 (sensitivity, 66.7%; specificity, 92.9%) (Figure 3b, Table 3).

## Discussion

This study aimed to investigate whether NWU, as an imaging index of brain edema before thrombectomy, predicts the functional prognosis of ischemic stroke patients in an extended time window of onset. We automatically measured NWU in patients with acute cerebral infarction in the anterior circulation using NECT-ASPECTS on admission. The primary findings of our study were as follows: NWU% based on NECTASPECTS could be used to predict 6h onset time point with an optimal cut-off value of 9.3. NWU% can be used as an independent predictor of the outcomes of patients who underwent thrombectomy within 6-24 hours from stroke onset to admission, and the best cutoff value was 13.3. A multiple regression model incorporating NWU, age, and ASPECTS predicted favorable functional outcomes at 90 days, with an AUC of 0.905.

Studies have shown that energy failure of nerve cells causes the cessation of sodium and potassium pumps and other intracellular energy-related activities during acute ischemia in the hyperacute phase of stroke^18^, ^19^. The net transfer of water from the outside to the inside of the cell leads to an increase in cell volume, reduction in extracellular space, and formation of cytotoxic edema^20^. As ischemia progresses, the blood-brain barrier is disrupted, vasogenic edema occurs on top of the original cytotoxic edema, and the diffusion of water molecules is further restricted^21^. Animal experiments in rats with MCA occlusion showed that the reduction in X-ray attenuation was inversely correlated with brain tissue water content, with each 1% increase in tissue water content corresponding to a reduction of 1.8 Hounsfield units (HU) in X-ray attenuation in the MCA region, and an average reduction of 6.2 HU in X-ray attenuation during the 6 hours MCA occlusion, indicating 3.4% water absorption^22^. Therefore, NWU can be used to detect brain edema and tissue lesions before thrombectomy. However, the current clinical time windows for intravenous thrombolysis and mechanical thrombectomy may not match the actual progression of brain tissue edema^23^. Previous studies on NWU have shown that tissue lesions are not very severe and may be suitable for thrombolysis, even if the stroke onset time is longer than 4.5 hours^24^. Some patients with onset beyond the recanalization time window and a mismatch in the imaging assessment, and the actual tissue lesions are already so severe that the prognosis is poor even with intravenous thrombolysis or mechanical retrieval of the thrombus. Our study thus aimed to investigate the ability of NWU based on NECT-ASPECTS to predict the onset time window in patients with acute anterior circulation ischemic stroke undergoing mechanical thrombectomy, as well as the functional outcome at 90 days.

First, we divided the patients into two groups according to the time from stroke onset to admission: 0-6 hours and 6-24 hours. The mean NWU for patients with onset times of 6-24 hours was 11.7, which was significantly higher than for patients with onset times of less than 6 hours. ROC analysis showed that the AUC of NWU% for predicting the onset time within 0-6 hours was 0.863, while the ideal cut-off value was 9.3. This is similar to the results of previous studies on stroke onset time using NWU% (9%-12%)^16^, ^24^. We further analyzed the distribution of NWU% between the two groups, finding that 19.2% of patients had NWU% less than 9.3 within 6-24 hours of stroke onset. This finding indicates that nearly 20% of the patients in the extended time window had milder edematous lesions. These patients between the 90-day functional outcomes may not need to spend more time evaluating advanced imaging and can proceed directly to recanalization.

We subsequently analyzed the factors influencing 90d mRS 3-6 in thrombectomy patients with a stroke onset time of 6-24 hours. The results of univariate regression analysis showed that age, ASPECT scores, onset time, and NWU% were important factors influencing the 90-day functional outcomes in patients who underwent thrombectomy within 6-24 hours (Table 2). Further multivariate regression analysis showed that age, ASPECT score, and NWU% may be independent factors influencing favorable functional outcomes at 90 days (Table 2). In addition, we found that the prediction of 90-day functional outcome by NWU was more sensitive in patients with an onset time of 6-24 hours compared to all patients with an onset time of 0-24 hours. In the early stages of ischemic stroke, neurotoxic edema forms within minutes of the interruption of cerebral blood flow^25^. Compared with nerve cells, brain interstitial cells (such as cerebral vascular endothelial cells) are more tolerant to ischemia^26^. The blood-brain barrier is destroyed after 3-4 hours, and vasogenic edema is formed^26^. The average onset time in the 0-6 hours group was 264.39 minutes, and approximately one-third of patients had an onset time within 4 hours. Therefore, we speculate that this statistical result may be due to the mild cerebral edema of the patient within 6 hours, as well as the lack of sensitivity to NWU recognition.

Finally, we performed ROC analysis in patients with an onset time within 6-24 hours, and the results showed that the optimal NWU% cut-off value for predicting good functional outcome at 90 days was 13.3, with an AUC of 0.856. The NWU% of patients with an mRS of 0-2 were all less than 13.3, while the NWU% of patients with mRS 3-6 was more than 13.3 in 61.1%. These results suggest that more than 50% of patients with pre-thrombectomy brain edema in the extended time window are already severely affected and may have unfavorable functional outcomes. Our results further showed that age, ASPECTS, and NWU% produced the best predictive models for good functional outcomes at 90 days, with the largest area under the ROC curve of 0.857, a sensitivity of 66.7%, and a specificity of 92.9%. NWU may predict higher intracranial pressure and higher arteriolar resistance, leading to a vicious cycle of interstitial pressure, worsening ischemic edema formation, and poor outcomes. Therefore, NWU may also serve as a therapeutic target to guide the clinical intensification of treatment to alleviate brain edema. Our results have a certain guiding significance and predictive value for the evaluation of pre-thrombectomy brain edema and functional outcomes in patients undergoing extended time window thrombectomy.

Our study had certain limitations. First, this was a retrospective study with a single-center design. Due to the strict inclusion criteria, our sample size was limited and subject to some bias. Our results will encourage further prospective multicenter studies to validate NWU as a broad alternative to advanced imaging mismatch. However, the optimal cut-off value of NWU% for our findings requires further randomized trials to verify the feasibility of NWU in guiding intravenous thrombolysis or endovascular thrombectomy in patients with an unknown time of symptom onset. Second, the automated software evaluation of NWU relies on AI-based software, which shows segmentation errors in patients with a deviated midline or incorrect posture, limiting its application.

## Conclusion

Quantification of lesion NWU can be used as an imaging biomarker reflecting the severity of pre-thrombectomy brain edema to identify and predict functional outcomes of patients with ischemic stroke within an extended time window.

## Figures and Tables

**Table 1:** Baseline characteristics of the study population

Characteristic	Patients with stroke within 6 hours	Patients with stroke within 6-24 hours	p
Demographics			
Subject, n (%)	56 (41.8%)	78 (58.2%)	—
Age, n (±SD)	59.2 (±12.5)	63.0 (±13.9)	0.196
Female, n (%)	18 (32.1%)	25 (32.1%)	0.982
Comorbidities			
Hypertension, n (%)	37 (66.1%)	48 (61.5%)	0.729
Diabetes mellitus, n (%)	11 (19.6%)	23 (29.5%)	0.008
Hypercholesterolemia, n (%)	9 (16.1%)	18 (23.1%)	0.025
Onset time, median (IQR)	264.4 (211-314)	621.2 (446-724)	—
Thrombectomy			
IVT, n (%)	16 (28.6%)	0	—
OTR, median (IQR)	337.41 (276-408.5)	698.99 (507-820)	0.000
Tirofiban, n (%)	39 (69.6%)	63 (80.8%)	0.068
NIHSS score, median (IQR)	14 (10-17)	15 (10-19)	0.269
NWU%, mean (±SD)	6.6 (±3.4)	11.7 (±3.0)	0.000
ASPECTS, median (IQR)	8 (7-9)	6 (5-8)	0.093
CTP mismatch, mean (±SD)	102.60 (±54.9)	96.4 (±70.7)	0.300
CBF<30%, median (IQR)	25.7 (3.6-44.7)	16.0 (0.5-20.7)	0.021
Site of arterial occlusion			0.257
ICA, n (%)	18 (32.1%)	18 (23.1%)	—
MCA-M1, n (%)	29 (51.7%)	45 (57.7%)	—
MCA-M2, n (%)	8 (14.3%)	12 (15.4%)	—
sICH, n (%)	5 (8.9%)	6 (7.7%)	—
mRS 3-6, n (%)	38 (67.9%)	40 (51.3%)	0.522

ASPECTS: Alberta Stroke Program Early Computed Tomography Score; Onset time: refer to the time from symptom onset to admission; IVT: Intravenous thrombolysis; OTR: onset-to-reperfusiontime; mTICI: NIHSS: National Institutes of Health Stroke Scale; NWU: net water uptake; CBF: Cerebral blood flow; CTP: CT perfusion.

**Table 2:** Logistic regression analysis to identify predictors of 90d mRS 3-6 in 6-24h group

Parameter	Univariable Analysis		Multivariable Analysis	
	OR (95%CI)	p	OR (95%CI)	p
Age	1.047 (1.009-1.086)	0.014	1.056 (1.002-1.112)	0.041
Female	0.880 (0.338-2.290)	0.793	—	—
NWU%	2.016 (1.455-2.792)	0.000	1.571 (1.030-2.396)	0.036
ASPECTS	0.420 (0.288-0.614)	0.000	0.554 (0.352-0.871)	0.011
Onset time	1.004 (1.001-1.007)	0.002	1.003 (1.000-1.007)	0.066
NIHSS	1.063 (0.976-1.159)	0.162	—	—
CTP mismatch	0.995 (0.988-1.002)	0.136	—	—
CBF<30%	1.002 (0.983-1.021)	0.847	—	—
Site of arterial occlusion	1.054 (0.743-1.479)	0.767		—

**Table 3:** NWU cut off for 6h onset time point and 90-day unfavorable functional outcome (mRS 3-6)

	Cut-off (%)	AUC (±SE)	Specificity	Sensitivity	95%CI	p
**6h**						
NWU%	9.3	0.863 (±0.03)	82.1%	80.8%	0.800-0.927	<0.001
**mRS (3-6)**						
NWU%	13.3	0.856 (±0.042)	100%	61.1%	0.774-0.939	<0.001
Age	—	0.695 (±0.062)	61.1%	71.4%	0.575-0.816	0.003
NWU%+Age	—	0.857 (±0.042)	92.9%	66.7%	0.774-0.940	<0.001
